# Short-term outcomes of virtual surgical planning–assisted mandibular reconstruction using a patient-specific reconstruction plate and split rib bundle bone graft: a prospective single-arm study

**DOI:** 10.1186/s40902-026-00520-0

**Published:** 2026-07-21

**Authors:** Mohamed Abdeldayem, Shady Hassan, Mariam Mohamed, Naglaa Shoukry

**Affiliations:** 1 Alexandria University, Alexandria, Egypt; 2 Suez University, Suez, Egypt

**Keywords:** Virtual surgical planning, Mandibular reconstruction, Patient-specific plate, Split rib bundle graft, Nonvascularized bone graft, Feasibility study, Benign mandibular lesions, Landmark-based analysis

## Abstract

**Background:**

Mandibular reconstruction requires restoration of continuity, contour, and occlusion to achieve acceptable functional and aesthetic outcomes. Virtual surgical planning (VSP) and patient-specific reconstruction plates may improve preoperative planning and intraoperative reproduction of mandibular alignment. This study evaluated the short-term outcomes of VSP-assisted mandibular reconstruction using a patient-specific reconstruction plate and split rib bundle bone graft (SRBG) in selected patients with benign mandibular lesions.

**Methods:**

This prospective single-arm clinical study included 30 adult patients with benign mandibular lesions requiring segmental mandibulectomy and immediate reconstruction. All patients underwent preoperative thin-slice computed tomography, virtual surgical planning, and fabrication of a patient-specific reconstruction plate. Reconstruction was performed using a split rib bundle graft. Clinical and radiographic follow-up was conducted for 12 months. Outcomes included mandibular symmetry, landmark-based radiographic discrepancy, graft-volume change, postoperative pain, occlusion, 12-month graft survival, patient satisfaction, and complications.

**Results:**

The mean age was 37.23 ± 10.15 years, and 56.7% of patients were male. Postoperative asymmetry indices were lower than preoperative values at all measured landmarks (all *p* < 0.001), with the overall mean asymmetry index decreasing from 41.91 ± 5.32 preoperatively to 6.48 ± 2.46 postoperatively. Landmark-based discrepancy between the virtual plan and postoperative CT was low, with menton deviation of 0.053 ± 0.266. Mean graft volume decreased significantly over time from 15.61 ± 2.22 cm³ at baseline to 14.11 ± 2.13 cm³ at 12 months (*p* < 0.001). Stable postoperative occlusion was achieved in 28 patients (93.3%). Twelve-month graft survival was observed in 29 of 30 patients (96.7%), and 76.7% of patients were very satisfied, while 13.3% were satisfied. Complications were uncommon; one patient (3.3%) developed late plate loosening/removal requiring secondary surgical intervention.

**Conclusion:**

VSP-assisted mandibular reconstruction using a patient-specific reconstruction plate and split rib bundle graft was feasible in this selected cohort of benign, nonirradiated mandibular defects, with acceptable short-term clinical and radiographic findings over 12 months. Because this was a single-arm study without a control group, with landmark-based radiographic assessment and limited follow-up, the results should be interpreted as descriptive feasibility data and should not be considered evidence of superiority, equivalence, or non-inferiority compared with conventional reconstruction, standard plates, or vascularized free-flap reconstruction.

## Introduction

Segmental mandibular defects produce major aesthetic and functional consequences, including loss of facial symmetry, impaired mastication, speech disturbance, altered swallowing, and disruption of occlusal relationships. Accordingly, mandibular reconstruction remains one of the most demanding procedures in maxillofacial surgery, because successful treatment requires not only restoration of continuity but also accurate recovery of contour, intermaxillary relationship, and the skeletal foundation needed for later oral rehabilitation. Historically, this process relied heavily on intraoperative freehand judgment, but the growing use of computer-assisted surgery has shifted much of the reconstructive decision-making from the operating room to the preoperative planning phase [1, 2].

Virtual surgical planning (VSP) and CAD/CAM technology have become increasingly integrated into mandibular reconstruction workflows because they allow three-dimensional analysis of the defect, simulation of osteotomies, predefinition of graft position, and fabrication of patient-specific guides and fixation hardware. This digital workflow is particularly attractive in complex mandibular defects, where even small errors in segment positioning may translate into malocclusion, asymmetry, condylar displacement, or difficulties with future prosthetic rehabilitation. Although microvascular bone flaps remain the standard reconstructive option for extensive composite, irradiated, or soft-tissue-deficient defects, VSP has been shown to improve operative efficiency and reproducibility, especially when combined with patient-specific plates [2–4].

Nonvascularized bone grafting maintains its utility in meticulously chosen mandibular defects. The technique of split rib bundle grafting is particularly noteworthy due to its capacity to provide a biologically viable corticocancellous graft that can be configured to restore mesiodistal span, vertical height, and buccolingual thickness. Furthermore, contemporary evidence supports the notion that nonvascularized grafts can yield significant success in segmental mandibular reconstruction, particularly in favorable recipient beds, especially among nonirradiated patients presenting with benign pathologies and sufficient soft-tissue coverage [5–7].

However, a gap remains between the modern digital reconstruction literature and the classical split rib bundle graft literature. Most contemporary VSP studies focus on fibula or other vascularized flaps, whereas the use of VSP and a custom-made mandibular plate to guide split rib bundle graft reconstruction has been much less thoroughly described. This distinction matters, because combining a low-technology biologic grafting concept with a high-precision digital workflow may offer a pragmatic alternative in selected benign mandibular defects, especially where prolonged microvascular surgery is unnecessary or impractical. Therefore, the present study aimed to describe the feasibility and 12-month clinical and radiographic outcomes of VSP-assisted mandibular reconstruction using a patient-specific reconstruction plate and split rib bundle bone graft in selected patients with benign mandibular defects [2–4].

## Patients and methods

### Study design and setting

This prospective single-arm clinical study was conducted on 30 adult patients diagnosed with benign mandibular lesions requiring segmental mandibulectomy and immediate reconstruction. All patients were managed at the Department of Cranio-Maxillofacial and Plastic Surgery, Faculty of Dentistry, Alexandria University, Egypt. The study was designed as a prospective single-arm feasibility and short-term outcomes study. No inference of comparative effectiveness, superiority, equivalence, or non-inferiority was intended.

The study was designed as a prospective single-arm evaluation. No concurrent control group was included. A matched historical control group was not used because previous cases did not have the same standardized VSP workflow, predefined radiographic landmark measurements, or serial CT-based graft-volume assessment. In addition, mandibular defects varied in site and extent, limiting the reliability of retrospective matching.

### Sample size

Sample size estimation was performed using G*Power software version 3.1.9.2 (Universität Kiel, Germany). Due to the exploratory single-arm design of the present study and the absence of directly comparable previous volumetric and landmark-based discrepancy data using patient-specific reconstruction plates with split rib bundle grafts, a pragmatic sample size approach was adopted.

The sample size was estimated based on previously reported postoperative complication rates in mandibular reconstruction studies, with a significance level (α) of 0.05 and study power of 80%. To compensate for possible dropout during follow-up, additional cases were included, resulting in a total sample size of 30 patients.

### Eligibility criteria

Patients were eligible if they were 18 years of age or older, of either sex, had a benign mandibular lesion planned for treatment by segmental mandibulectomy, and were expected to have adequate soft-tissue coverage after resection. Patients were excluded if they were medically unfit for general anesthesia, had soft-tissue deficiency better managed by free osseocutaneous flap reconstruction, had a history of head and neck radiotherapy or were scheduled to receive postoperative radiotherapy, had obvious preoperative infection at the resection site, or had recurrent lesions requiring wider composite resection and free flap reconstruction.

### Preoperative assessment

All patients underwent detailed history taking and full clinical examination. Preoperative assessment included evaluation of lesion characteristics, previous surgical history, medical comorbidities, and intraoral status, particularly oral hygiene and dental condition as potential contributors to postoperative infection. Standardized preoperative photographs were obtained. Routine laboratory investigations included complete blood count, blood group, bleeding and clotting profile, fasting blood glucose, liver function tests, and renal function tests. Before surgery, the oral cavity was meticulously prepared by scaling, elimination of septic foci, restoration of carious teeth when indicated, treatment of gingival or periodontal infection, and removal of retained roots.

### Virtual surgical planning

Preoperative thin-slice maxillofacial computed tomography was obtained for all patients and converted into a three-dimensional digital mandibular model. Virtual surgical planning was performed to define the extent of resection, determine the osteotomy lines, restore mandibular contour, and plan the intended postoperative mandibular alignment. The design process was performed using Mimics and 3-matic software (Materialise NV, Leuven, Belgium).

The patient-specific reconstruction plate was designed by a member of the surgical team experienced in virtual surgical planning. The design was based on preoperative CT data and the virtual reconstruction plan, with the aim of improving plate adaptation to the planned mandibular contour and reducing the need for intraoperative manual plate bending.

Using Mimics and 3-matic software, segmentation was performed to delineate the tumor boundaries and define the resection margins. A mirroring tool was then used to design the contour of the reconstruction plate so that it reproduced the native mandibular contour. The plate was manufactured from medical-grade titanium (Grade 4) using a milling technique, with a final thickness of 1.5 mm.

**Fig. 1:**
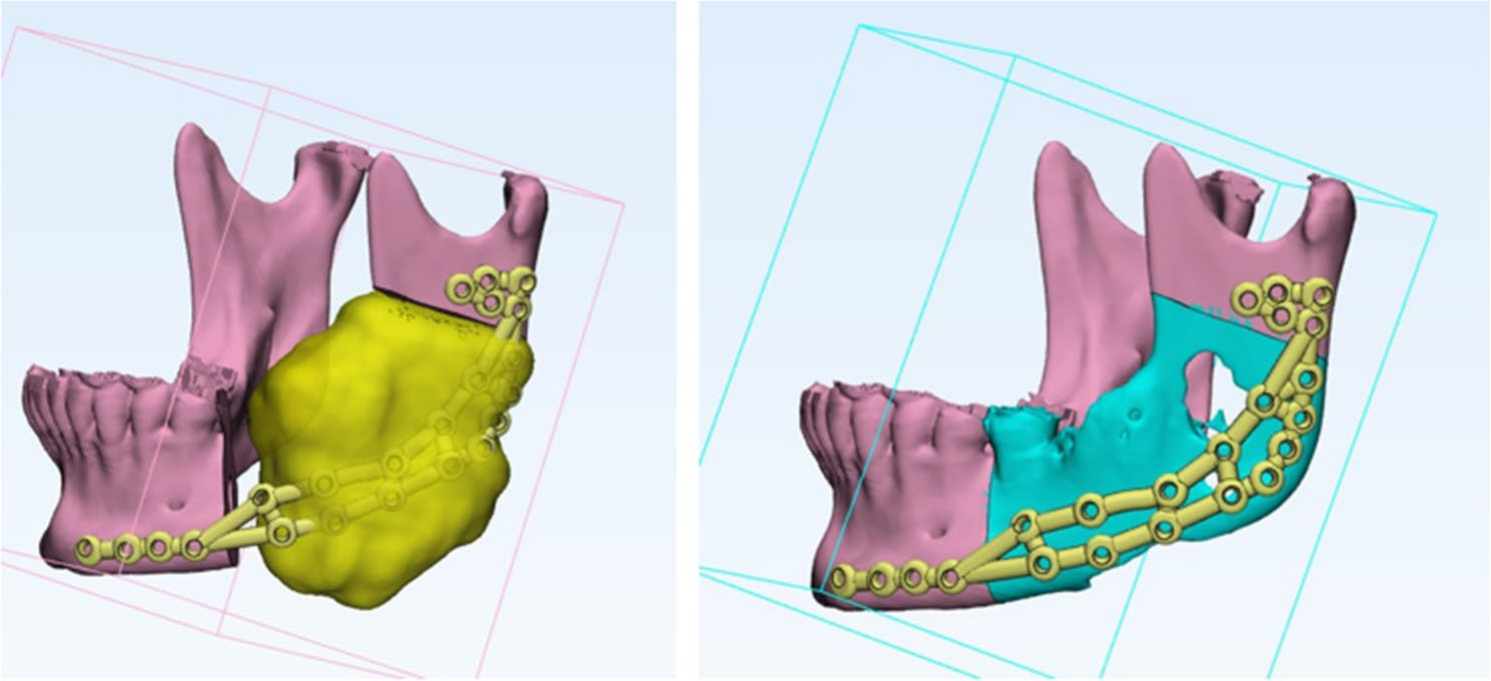
Computer assisted 3D reconstruction of mandible and custom designed plate

In the present cohort, intraoperative transfer of the virtual plan relied primarily on the patient-specific reconstruction plate and intraoperative anatomical alignment. Separate cutting guides, drilling guides, positioning guides, or rib-harvest guides were not described in the present protocol. Therefore, the workflow should be interpreted as VSP-assisted reconstruction using a patient-specific plate rather than a fully guide-based CAD/CAM reconstruction protocol (Fig. 1).

### Surgical technique

All procedures were performed under general anesthesia. Surgical access was selected according to defect location. Defects involving the mandibular body, angle, and ramus were approached through a combined intraoral lower sulcus incision and extraoral extended submandibular incision when required. When the lesion extended to the mandibular angle, coronoidectomy was performed to minimize displacement of the proximal segment caused by temporalis muscle traction. In cases where the lesion approximated or involved the condyle, disarticulation was performed with preservation of the articular disc whenever feasible.

**Fig. 2:**
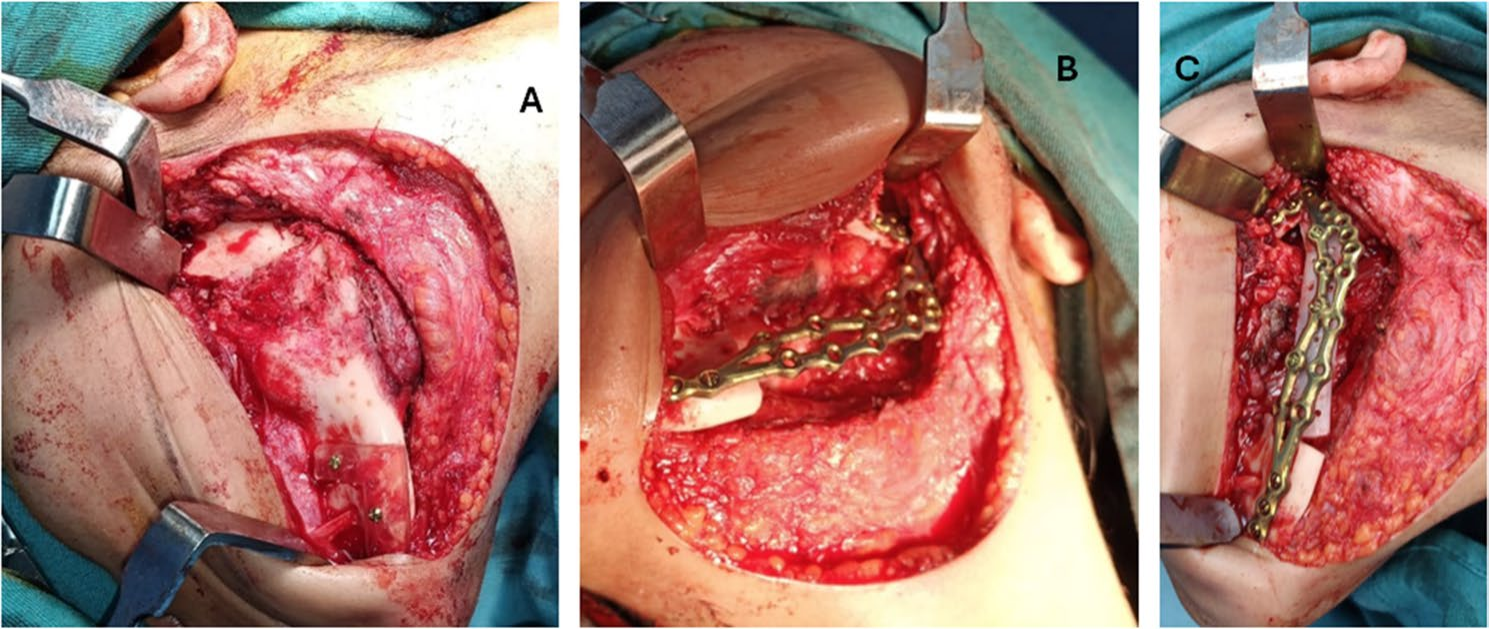
**A **Surgical incision of mandible showing tumour, **B** Surgical site after excision of tumor and making sure custom plate is aligned, **C** Final alignment of custom plate and Rib graft

After resection, the proximal and distal mandibular stumps were contoured and smoothed to facilitate tension-free watertight mucosal closure and to prevent mucosal perforation by sharp bony edges. The outer cortex at the recipient sites was decorticated using a rose-head bur to expose cancellous bone and improve graft incorporation. Rib grafts were harvested through a curved submammary incision, usually from two alternating ribs. When condylar reconstruction was required, a costochondral component was preserved. After confirmation of pleural integrity, the donor wound was closed in layers. The harvested ribs were then split longitudinally using a fine osteotome while preserving the medullary component (Fig. 2).

The patient-specific reconstruction plate was first fixed to the residual mandibular stumps using at least three screws on each side of the defect. One split rib segment was then fixed proximally and another distally within the prepared recipient bed. The remaining rib segments were telescoped to bridge the defect and secured together as a bundle using screws, with or without transosseous circumferential wires, and then fixed to the reconstruction plate. Additional rib segments were added when necessary to restore buccolingual thickness and vertical height while preserving adequate interarch space for future dental rehabilitation. In cases of condylar resection, a split costochondral graft was used for condylar replacement. The oral mucosa was closed in two layers using Vicryl sutures, and the external wound was closed in three layers with suction drainage maintained for 2 to 3 days.

Graft shaping and fixation time was defined as the interval beginning immediately after rib graft harvesting and extending through graft shaping, adaptation to the patient-specific reconstruction plate, and fixation to the mandibular defect. This variable did not include tumor resection, surgical exposure, or rib harvesting and therefore should not be interpreted as total operative time.

### Outcome assessment and follow-up

Suction drainage was maintained for 2 to 3 days after surgery. Patients were followed clinically and radiographically for 12 months. CT-based graft-volume assessment was performed at the immediate postoperative baseline, 6 months, and 12 months. No formal postoperative physiotherapy or occlusal-guidance protocol was routinely required during follow-up, as satisfactory clinical occlusion and functional stability were achieved without additional occlusal rehabilitation measures in the studied cohort.

Stable postoperative occlusion was defined clinically as maintenance of a reproducible postoperative occlusal relationship without frank malocclusion, malocclusion relapse, or need for additional occlusal rehabilitation during follow-up. Minor occlusal discrepancy referred to a clinically detectable occlusal irregularity that did not require secondary surgical correction and was not classified as postoperative malocclusion.

Patient satisfaction was assessed using a simple five-point Likert scale: very satisfied, satisfied, neutral, dissatisfied, or very dissatisfied. No validated quality-of-life or patient-reported outcome instrument, such as the University of Washington Quality of Life questionnaire or FACE-Q, was used.

Twelve-month graft survival was defined as maintenance of graft continuity and immobilization without graft exposure, graft removal, or secondary surgical intervention for graft-related failure during the 12-month follow-up period.

Mandibular symmetry was assessed using a coordinate-based asymmetry index (AI), as described by Huang et al. The AI was calculated for each selected landmark using the following equation:

$$\begin{aligned} \mathrm{AI}&=\surd\left[\left(\mathrm{LdS-RdS}\right)^{2}+\left(\mathrm{LdA-RdA}\right)^{2}\right. \\& \left.+\left(\mathrm{LdC-RdC}\right)^{2}\right] \end{aligned}$$

where L and R represent the left and right sides, respectively, and S, A, and C represent sagittal, axial, and coronal coordinates. The AI is a linear coordinate-based index and was reported in millimeters. Lower AI values indicate greater symmetry. The landmarks included condylion (Co), defined as the most superior point on the mandibular condyle; gonion (Go), defined as the most posteroinferior point at the mandibular angle; mental foramen (Mf), defined as the center of the mental foramen, Menton (Me) was defined as the most inferior midline point of the mandibular symphysis on the three-dimensional mandibular model; and La, defined as the lowest point on the mandibular lower border at the angle region.

Graft volume was assessed using CT-based three-dimensional segmentation analysis. DICOM data obtained from postoperative CT scans were imported into Mimics software (Materialise NV, Leuven, Belgium). The grafted mandibular segment was segmented semi-automatically using threshold-based bone selection followed by manual refinement when required. Three-dimensional reconstruction of the segmented graft was then generated, and volumetric measurements (cm³) were automatically calculated by the software at immediate postoperative, 6-month, and 12-month follow-up intervals. Radiographic discrepancy was assessed using a predefined landmark-based approach. The values reported in Table 1 represent landmark-based discrepancy measurements between the planned and postoperative mandibular positions. Surface-based three-dimensional registration, mean surface deviation, and root-mean-square error analysis were not performed because the study protocol was based on anatomical landmark measurements and serial CT-based volumetric assessment. Therefore, these data should be interpreted as landmark-based discrepancy measurements rather than full surface-based three-dimensional accuracy analysis.

**Table 1:** Landmark-based radiographic discrepancy outcomes

Radiographic discrepancy variable	Mean ± SD	Range
Co reconstructed side	0.171 ± 0.262	-0.30 to 0.70
Co healthy side	0.058 ± 0.158	-0.18 to 0.34
La reconstructed side	0.190 ± 0.359	-0.20 to 1.06
La healthy side	0.076 ± 0.268	-0.50 to 0.60
Go reconstructed side	0.004 ± 0.151	-0.30 to 0.30
Go healthy side	0.229 ± 0.383	-0.20 to 1.10
Mf reconstructed side	-0.003 ± 0.255	-0.60 to 0.30
Mf healthy side	0.123 ± 0.376	-0.50 to 0.90
Menton	0.053 ± 0.266	-0.60 to 0.40

Radiographic measurements were performed by a single examiner using a predefined landmark-based protocol. The examiner was not fully blinded because the reconstruction plate and graft were visible on postoperative CT images. To reduce bias, CT datasets were anonymized and reviewed in randomized order without clinical identifiers. Intra-examiner reliability was assessed and demonstrated excellent agreement, with intraclass correlation coefficient values ≥ 0.99. Inter-observer reliability was not assessed. Therefore, the radiographic findings should be interpreted as single-examiner landmark-based measurements rather than fully blinded or independently replicated three-dimensional accuracy data.

### Statistical analysis

All statistical analyses were performed using IBM SPSS Statistics. Continuous variables were summarized as mean ± standard deviation (SD), whereas categorical variables were presented as frequencies and percentages. Preoperative and postoperative asymmetry indices were compared using paired-samples t-tests. Because multiple paired landmark comparisons were performed, Holm-Bonferroni correction was applied to the asymmetry-index analyses. Serial graft-volume measurements at baseline, 6 months, and 12 months were analyzed using repeated-measures analysis of variance. Because Mauchly’s test indicated violation of the sphericity assumption, Greenhouse-Geisser-corrected results were reported. Postoperative pain across follow-up time points was analyzed using Friedman’s test followed by Bonferroni-adjusted pairwise comparisons. Comparisons according to defect site were performed using one-way analysis of variance for continuous variables and categorical testing as appropriate. When overall between-group graft-volume comparisons were significant, Bonferroni post hoc testing was used. No formal multiplicity correction was applied across all different outcome domains; therefore, secondary analyses were interpreted as exploratory.

## Results

### Patient characteristics

A total of 30 patients were included in the study. The mean age was 37.23 ± 10.15 years. Seventeen patients (56.7%) were male and 13 (43.3%) were female. The most common pathology was ameloblastoma, identified in 12 patients (40.0%), followed by odontogenic keratocyst in 8 (26.7%), odontogenic myxoma in 4 (13.3%), vascular malformation in 3 (10.0%), and ossifying fibroma in 3 (10.0%).

For descriptive reporting, mandibular defects were grouped according to anatomical extent into body defects, body and angle defects, and body, angle, and ramus defects (Table 2). A formal validated mandibular defect classification system was not applied. .Regarding defect location, 13 patients (43.3%) had body defects, 9 (30.0%) had body plus angle defects, and 8 (26.7%) had body plus angle plus ramus defects. The mean graft shaping and fixation time for the entire cohort was 59.13 ± 12.25 min (Table 3).

**Table 2:** Anatomical classification of mandibular defects included in the study

Number of Cases (*n*=30)	Anatomical Extent	Defect Classification
13	Body	Lateral mandibular defects
9	Body + Angle	Extended lateral mandibular defects
8	Body + Angle + Ramus	Hermimandibular defects

**Table 3:** Baseline demographic and clinical characteristics of the study cohort

Variable	*n* (%) or mean ± SD *n* = 30
Age (years)	37.23 ± 10.15
Sex	
Male	17 (56.7)
Female	13 (43.3)
Etiology / tumour type (histology)	
Ameloblastoma	12 (40.0)
Odontogenic keratocyst	8 (26.7)
Odontogenic myxoma	4 (13.3)
Vascular malformation	3 (10.0)
Ossifying fibroma	3 (10.0)
Defect site	
Body defect	13 (43.3)
Body + angle defect	9 (30.0)
Body + angle + ramus defect	8 (26.7)
Graft Shaping and fixation time (min)	59.13 ± 12.25

### Symmetry indices and landmark-based radiographic discrepancy

**Fig. 3:**
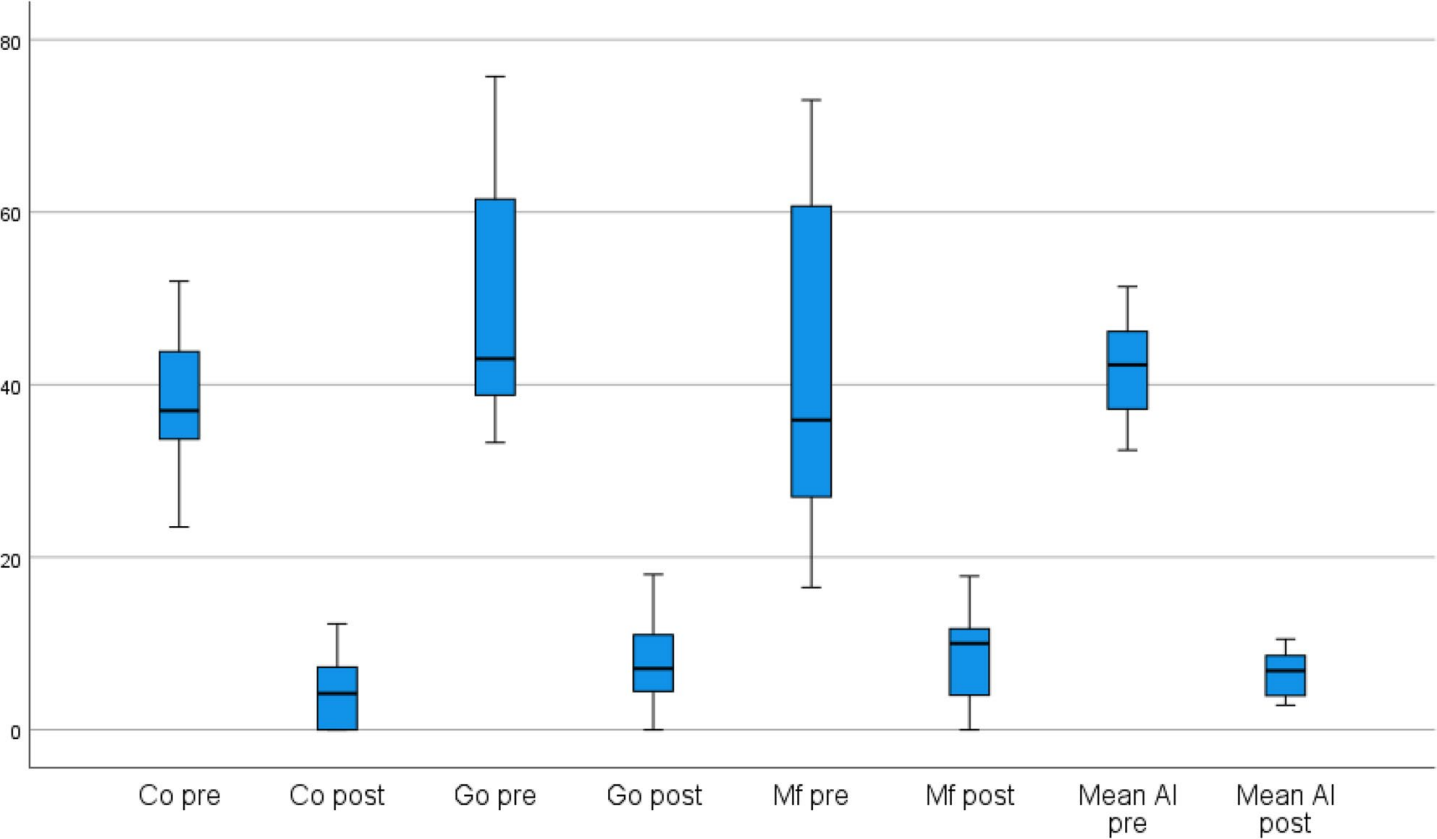
Preoperative and postoperative symmetry indices

Postoperative asymmetry indices were lower than preoperative values at all measured landmarks (Table 4, Fig. 3). The Co asymmetry index decreased from 37.99 ± 8.37 preoperatively to 4.60 ± 4.12 postoperatively, corresponding to a mean difference of 33.39 (95% CI 29.69 to 37.10; *p* < 0.001). Similarly, the La asymmetry index decreased from 38.90 ± 9.48 to 4.51 ± 4.36, with a mean difference of 34.39 (95% CI 31.70 to 37.10; *p* < 0.001). The Go asymmetry index decreased from 49.33 ± 14.32 to 8.38 ± 5.21, with a mean difference of 40.94 (95% CI 36.20 to 45.70; *p* < 0.001). The Mf asymmetry index decreased from 41.41 ± 18.05 to 8.42 ± 5.53, with a mean difference of 32.99 (95% CI 26.80 to 39.10; *p* < 0.001). The overall mean asymmetry index declined from 41.91 ± 5.32 preoperatively to 6.48 ± 2.46 postoperatively, corresponding to a mean difference of 35.43 (95% CI 33.63 to 37.23; *p* < 0.001) (Table 4, Fig. 3).

**Table 4:** Symmetry assessment outcomes before and after reconstruction

Variable	Preoperative mean ± SD	Postoperative mean ± SD	MD (95% CI)	*p* value
Co asymmetry index	37.99 ± 8.37	4.60 ± 4.12	33.39 (29.69 to 37.10)	**< 0.001**
La asymmetry index	38.90 ± 9.48	4.51 ± 4.36	34.39 (31.70 to 37.10)	**< 0.001**
Go asymmetry index	49.33 ± 14.32	8.38 ± 5.21	40.94 (36.20 to 45.70)	**< 0.001**
Mf asymmetry index	41.41 ± 18.05	8.42 ± 5.53	32.99 (26.80 to 39.10)	**< 0.001**
Overall mean asymmetry index	41.91 ± 5.32	6.48 ± 2.46	35.43 (33.63 to 37.23)	**< 0.001**

Landmark-based radiographic discrepancy analysis showed low mean deviations between the virtual plan and postoperative CT measurements (Table 1). Mean discrepancy values ranged from − 0.003 ± 0.255 at the reconstructed-side Mf to 0.229 ± 0.383 at the healthy-side Go. Mean menton deviation was 0.053 ± 0.266. Because the analysis was landmark-based and performed by a single non-blinded examiner, these findings should not be interpreted as surface-based three-dimensional accuracy data.

### Operative and clinical outcomes according to defect site

The mean graft shaping and fixation time was 59.13 ± 12.25 min (Table 3). Patient satisfaction by defect site also did not differ significantly (*p* = 0.383) (Table 5). In the body plus angle plus ramus group, all patients were very satisfied (100.0%). In the body plus angle group, 7 patients (77.8%) were very satisfied, 1 (11.1%) was satisfied, and 1 (11.1%) was neutral. In the body group, 8 patients (61.5%) were very satisfied, 3 (23.1%) were satisfied, and 2 (15.4%) were neutral.

**Table 5:** Operative and clinical outcomes according to defect site

Variable	Body + Angle + Ramus (*n* = 8)	Body + Angle (*n* = 9)	Body (*n* = 13)	Total (*n* = 30)	*p* value
Graft shaping and fixation time (min)	64.88 ± 5.96	62.33 ± 17.23	53.38 ± 8.86	59.13 ± 12.25	0.068
Patient satisfaction, n (%)					
Very satisfied	8 (100.0)	7 (77.8)	8 (61.5)	23 (76.7)	0.383^*^
Satisfied	0 (0.0)	1 (11.1)	3 (23.1)	4 (13.3)	
Neutral	0 (0.0)	1 (11.1)	2 (15.4)	3 (10.0)	

### Graft-volume changes and postoperative pain

**Fig. 4:**
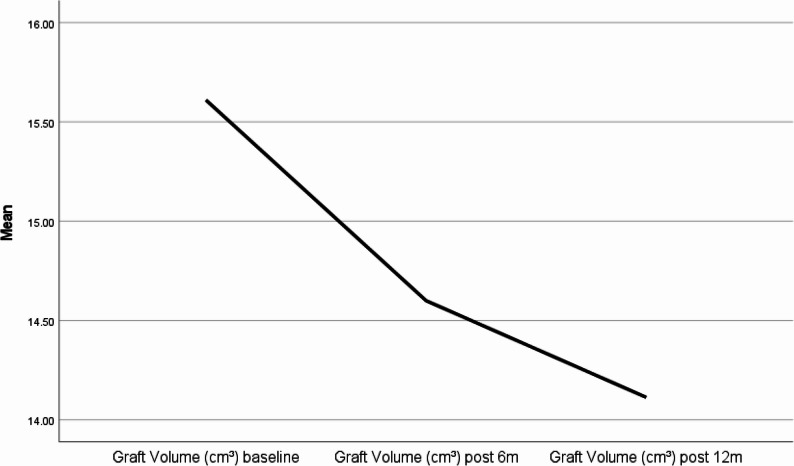
Graft-volume change over time

Graft volume progressively decreased during follow-up (Table 6, Fig. 4). For the whole cohort, the mean graft volume declined from 15.61 ± 2.22 cm³ at baseline to 14.60 ± 2.10 cm³ at 6 months and 14.11 ± 2.13 cm³ at 12 months. Repeated-measures analysis of variance demonstrated a significant effect of time on graft volume, which remained statistically significant after Greenhouse-Geisser correction (F = 100.304, *p* < 0.001) (Table 6, Fig. 4).

**Table 6:** Graft volume according to defect site over time and postoperative pain outcomes

Time point	Body + Angle + Ramus (*n* = 8)	Body + Angle (*n* = 9)	Body (*n* = 13)	Total (*n* = 30)	*p* value
Baseline graft volume (cm³)	18.50 ± 0.69	16.04 ± 0.73	13.53 ± 0.96	15.61 ± 2.22	**< 0.001**
Postoperative 6-month graft volume (cm³)	17.29 ± 0.58	15.10 ± 0.71	12.60 ± 0.88	14.60 ± 2.10	**< 0.001**
Postoperative 12-month graft volume (cm³)	16.86 ± 0.55	14.47 ± 0.83	12.18 ± 1.09	14.11 ± 2.13	**< 0.001**
Repeated-measures ANOVA, Greenhouse-Geisser F	F = 100.304				**< 0.001**

Post operative pain at donor and recipient site Bonferroni-adjusted pairwise pain comparisons					
Comparison	**Adjusted** ***p*** **value**				
2 weeks vs. 1 week	**0.008**				
2 weeks vs. 24 h	**< 0.001**				
2 weeks vs. preoperative	**< 0.001**				
1 week vs. 24 h	0.056				
1 week vs. preoperative	**< 0.001**				
24 h vs. preoperative	**0.008**				
Friedman test for pain, χ²	88.946				
Friedman test p value	**< 0.001**				

When analyzed according to defect site, graft volume differed significantly among groups at all follow-up time points (Table 6). At baseline, mean graft volumes were 18.50 ± 0.69 cm³ in the body plus angle plus ramus group, 16.04 ± 0.73 cm³ in the body plus angle group, and 13.53 ± 0.96 cm³ in the body group (*p* < 0.001). At 6 months, the corresponding values were 17.29 ± 0.58 cm³, 15.10 ± 0.71 cm³, and 12.60 ± 0.88 cm³, respectively (*p* < 0.001). At 12 months, mean graft volume remained highest in the body plus angle plus ramus group (16.86 ± 0.55 cm³), followed by the body plus angle group (14.47 ± 0.83 cm³) and the body group (12.18 ± 1.09 cm³) (*p* < 0.001). Bonferroni post hoc analysis showed significant pairwise differences between all defect-site groups at each time point.

Postoperative pain also changed significantly over time (Table 6). Friedman’s test demonstrated a significant overall difference in pain scores across the assessed time points (χ² = 88.946, *p* < 0.001). Bonferroni-adjusted pairwise comparisons showed significant differences between 2 weeks and 1 week (adjusted *p* = 0.008), 2 weeks and 24 h (adjusted *p* < 0.001), 2 weeks and preoperative assessment (adjusted *p* < 0.001), 1 week and preoperative assessment (adjusted *p* < 0.001), and 24 h and preoperative assessment (adjusted *p* = 0.008). The comparison between 1 week and 24 h did not remain statistically significant after adjustment (adjusted *p* = 0.056).

### Functional outcomes, 12-month graft survival, and complications

**Fig. 5:**
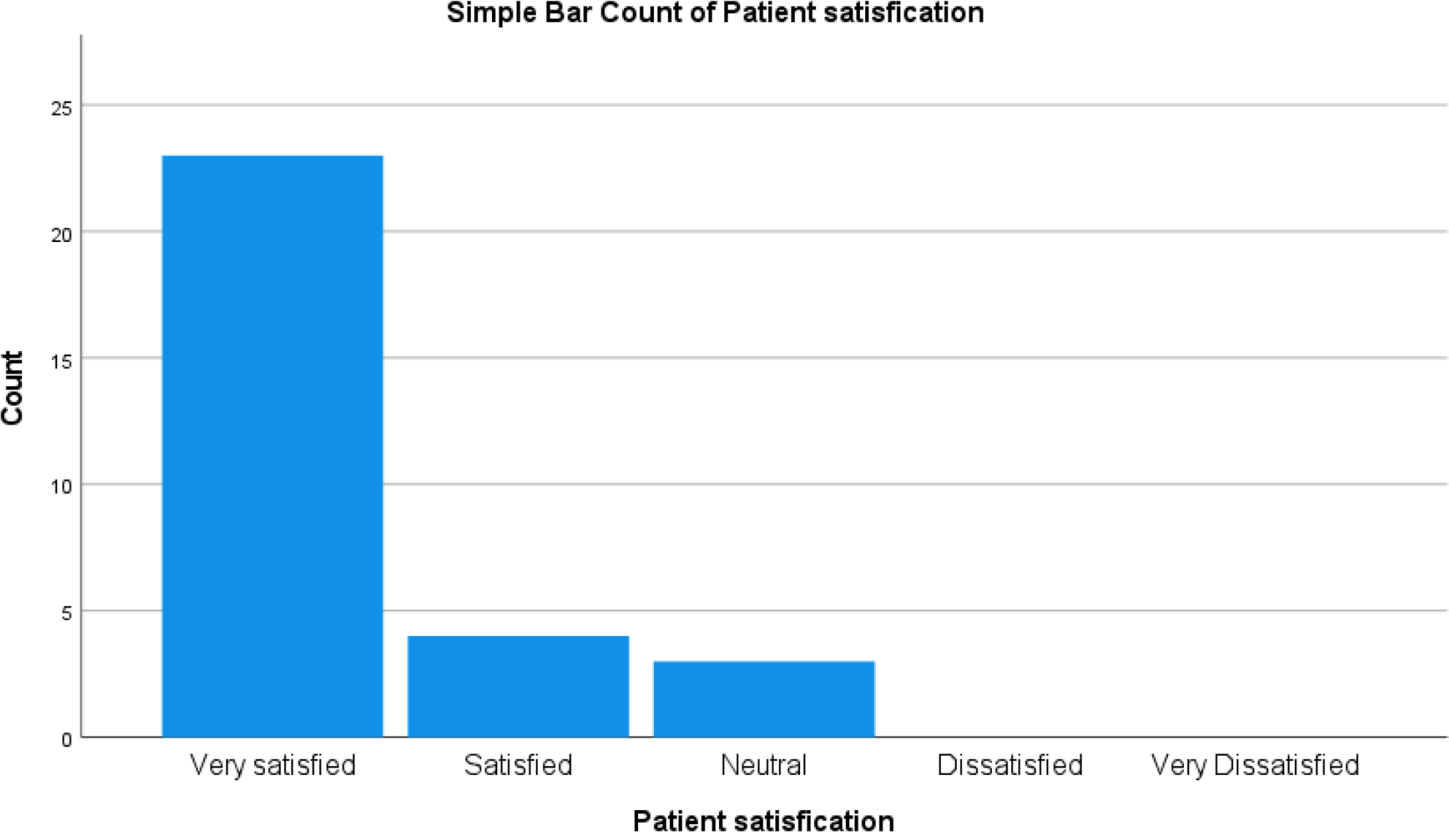
Patient satisfaction

Functional outcomes were favorable overall (Table 7, Fig. 5). Stable postoperative occlusion was achieved in 28 patients (93.3%), whereas 2 patients (6.7%) had a minor occlusal discrepancy. No cases of postoperative malocclusion were recorded. Likert-scale satisfaction ratings were favorable: 23 patients (76.7%) were very satisfied, 4 (13.3%) were satisfied, and 3 (10.0%) were neutral, with no patients reporting dissatisfaction or very poor satisfaction.

**Table 7:** Functional outcomes, 12-month graft survival, and postoperative complications

Outcome	*n* (%)
Postoperative occlusion	
Stable postoperative occlusion	28 (93.3)
Minor occlusal discrepancy	2 (6.7)
Malocclusion	0 (0.0)
Patient satisfaction	
Very satisfied	23 (76.7)
Satisfied	4 (13.3)
Neutral	3 (10.0)
Dissatisfied	0 (0.0)
Very dissatisfied	0 (0.0)
Twelve-month graft survival	
Successful integration and immobilization	29 (96.7)
Absence of exposure	29 (96.7)
Secondary surgical intervention/reoperation	1 (3.3)
Overall 12-month graft survival	29 (96.7)
Overall 12-month graft failure	1 (3.3)
Postoperative complications	
Recipient-site infection	0 (0.0)
Recipient-site wound dehiscence	0 (0.0)
Plate loosening requiring removal	1 (3.3)
Secondary surgical intervention/reoperation	1 (3.3)
Malocclusion relapse	0 (0.0)
Donor-site infection	0 (0.0)
Donor-site wound dehiscence	0 (0.0)
Pneumothorax	0 (0.0)
Early complication during first 6 months	0 (0.0)
Late complication during second 6 months	1 (3.3)

Twelve-month graft survival was observed in 29 of 30 patients (96.7%) (Table 7). Graft integration and immobilization at 12 months were documented in 29 patients (96.7%), and absence of graft exposure was observed in 29 patients (96.7%). One patient (3.3%) developed late plate loosening/removal requiring secondary surgical intervention; this event was counted as a late complication and reoperation. No recipient-site infection, recipient-site wound dehiscence, malocclusion relapse, donor-site infection, donor-site wound dehiscence, pneumothorax, or early complication during the first 6 months was observed (Table 7).

## Discussion

### Principal interpretation of the present findings

The present study describes the feasibility and short-term clinical performance of VSP-assisted mandibular reconstruction using a patient-specific reconstruction plate and split rib bundle graft in selected patients with benign mandibular lesions. Postoperative asymmetry indices were lower than preoperative values, and landmark-based discrepancy values between the planned and postoperative mandibular positions were low. These findings were accompanied by stable occlusion in most patients, 12-month graft survival in 29 of 30 patients, low observed complication frequency, and favorable satisfaction ratings on a non-validated Likert scale.

However, the findings should be interpreted within the limits of the study design. This was a prospective single-arm study without a conventional reconstruction, standard plate, non-VSP rib graft, or vascularized flap comparator. Therefore, the results demonstrate feasibility and short-term descriptive outcomes in selected cases, but they do not establish superiority, equivalence, or non-inferiority compared with other reconstructive approaches.

### Context with existing literature

Existing mandibular reconstruction studies using VSP and patient-specific plates have most commonly focused on vascularized fibula or other osseous free-flap reconstruction. Several systematic reviews and clinical series have reported improved surgical planning, flap positioning, and postoperative mandibular contour reproduction when VSP is incorporated into fibula free-flap reconstruction [3, 8–10]. In contrast, the present study evaluated a selected cohort treated with a nonvascularized split rib bundle graft. Therefore, direct comparison with vascularized flap series is limited by differences in indication, defect complexity, soft-tissue requirements, graft biology, and outcome assessment methods. The present findings are best viewed as short-term descriptive data from a selected benign, nonirradiated cohort rather than evidence that this technique is equivalent or superior to conventional reconstruction, standard plates, or vascularized free-flap reconstruction. Similar caution has been emphasized in reviews comparing vascularized and nonvascularized mandibular reconstruction techniques, where patient selection and defect characteristics substantially influence outcomes [11, 12].

### Potential role of the digital workflow

The digital workflow may have helped standardize preoperative three-dimensional assessment of the defect, planned mandibular contour restoration, and fabrication of a plate adapted to the intended reconstruction. In mandibular reconstruction, errors in segment positioning can affect facial symmetry, mandibular contour, condylar position, and occlusal relationships. In the present study, VSP was used to shift part of this decision-making from intraoperative estimation to preoperative planning [4, 13].

Nevertheless, the workflow should not be overstated. The present protocol relied primarily on VSP-assisted planning and a patient-specific reconstruction plate; separate cutting, drilling, positioning, or rib-harvest guides were not described. Therefore, the findings are best interpreted as outcomes of a plate-assisted VSP workflow rather than a fully guide-based digital reconstruction protocol.

The radiographic findings of the current study align well with this broader literature. Roser et al. showed early on that free fibula mandibular reconstruction planned virtually could closely reproduce the intended surgical result, while Barr et al., in their systematic review and meta-analysis, concluded that VSP improves operative efficiency and may improve accuracy and outcomes compared with traditional techniques. More recently, El-Mahallawy et al. reported a highly significant degree of agreement between preoperative virtual planning and postoperative outcomes and also emphasized the need for standardized evaluation methods when accuracy is assessed. The present study adds descriptive short-term data in a different reconstructive context: a nonvascularized rib-graft construct stabilized with a patient-specific plate rather than a fibula free-flap series. This raises the possibility that VSP-assisted plate adaptation may also be useful in selected nonvascularized graft reconstructions, although this cannot be confirmed without a comparative study and surface-based three-dimensional accuracy analysis [3, 4, 13].

The patient-specific reconstruction plate may have facilitated intraoperative reproduction of the planned mandibular contour by providing a precontoured fixation framework and limiting the need for intraoperative manual plate adaptation. In the present series, the low landmark-based discrepancy values and favorable occlusal outcomes are consistent with this mechanism. However, because no standard-plate control group was included, the incremental benefit of the patient-specific plate cannot be isolated from the effects of careful case selection, surgical technique, and graft fixation [14–16].

### Biological plausibility and performance of the split rib bundle graft

The present study also revisits a classical biologic reconstructive concept through a modern planning framework. The split rib bundle graft has established historical use in mandibular reconstruction because it can provide mesiodistal span, vertical height, and buccolingual thickness through telescoping and augmentation of split rib segments. The split rib exposes cancellous and marrow-containing surfaces and allows a compact graft architecture that can be adapted to the defect. In the present study, this biologic grafting concept was combined with preoperative digital planning and patient-specific plate fixation [2, 6].

In the present cohort, graft volume decreased significantly over time, yet this occurred alongside 12-month graft survival in 29 of 30 patients (96.7%), stable postoperative occlusion in 93.3% of patients, and one case requiring secondary surgical intervention for plate loosening/removal. Although the exact cause of this complication could not be definitively identified from the available data, poor oral hygiene and inadequate compliance with postoperative maintenance were considered possible contributing factors. Other factors, including graft remodeling, local bone quality, fixation loading, and surgical variables, may also have contributed. This event has been reported consistently as a late complication and reoperation in the revised Results and Table 7.

Graft volume reduction should be interpreted cautiously. The observed decrease may represent early postoperative remodeling of the nonvascularized rib graft; however, the present 12-month follow-up period is insufficient to determine whether this change stabilizes over time or represents progressive resorption. Importantly, the volume reduction did not appear to compromise mandibular continuity or clinical stability in most patients during the first postoperative year. Longer follow-up is required to evaluate whether graft volume remains stable beyond 12 months and whether late mechanical or biological complications emerge [17, 18].

These favorable results should also be interpreted in light of patient selection. The inclusion criteria deliberately focused on benign lesions with adequate soft-tissue coverage and excluded patients with infection, previous or planned radiotherapy, soft-tissue deficiency requiring free osseocutaneous flap reconstruction, and recurrent lesions requiring wider composite resection. These exclusions were not incidental; they define the clinical setting in which nonvascularized grafting was considered biologically reasonable. Therefore, the observed 12-month graft survival should be interpreted as evidence of feasibility in appropriately selected cases rather than evidence that vascularized reconstruction is unnecessary [5, 19].

### Functional recovery, esthetics, and the patient perspective

From a clinical perspective, mandibular reconstruction should be judged not only by radiographic discrepancy but also by facial symmetry, oral competence, comfort, occlusal stability, and absence of major complications requiring reintervention. In this regard, the present findings are encouraging. Stable occlusion was achieved in nearly all patients, frank postoperative malocclusion was not observed. Likert-scale satisfaction ratings were favorable across defect categories, These findings indicate acceptable short-term clinical and patient-reported observations in this cohort, although the absence of a validated quality-of-life instrument limits interpretation.

These patient-centered outcomes are also conceptually aligned with contemporary reconstructive priorities, which emphasize restoration of mandibular arch form, preservation of intermaxillary relationships, and maintenance of a skeletal foundation suitable for later oral rehabilitation. Although the study did not evaluate implant placement or final dental rehabilitation, the use of a patient-specific reconstruction plate, restoration of symmetry, and preservation of interarch space suggest that future rehabilitation was considered during reconstruction. This point should be interpreted cautiously because prosthetic or implant-based outcomes were not assessed [2, 4, 20].

The postoperative pain profile and donor-site data add another layer to this interpretation. Pain declined significantly over follow-up, and no donor-site infection, dehiscence, or pneumothorax was reported. Rib grafting is often viewed with caution because of concerns about donor morbidity, but these results suggest that, with careful technique and case selection, donor-site complications can remain low. That observation is consistent with the traditional appeal of costal grafting as a less resource-intensive option with acceptable morbidity in selected patients. Because uncommon donor-site complications may be underdetected in small series, the present findings should be interpreted as showing low observed donor-site morbidity in this cohort rather than proving minimal donor morbidity [18].

### Where this technique fits within current reconstructive practice

The present findings should be positioned within a selective reconstructive framework. Microvascular osseous free flaps remain the standard reconstructive option for extensive segmental defects, composite defects, irradiated beds, soft-tissue-deficient defects, malignant defects requiring wider ablation, and cases requiring large-volume tissue replacement. The present technique should therefore not be interpreted as a replacement for vascularized reconstruction. This interpretation remains hypothesis-generating because the study did not include a conventional non-VSP rib graft, standard plate, or vascularized free-flap comparator [4, 21].

The possible practical value of this approach is relevant, particularly in settings where the duration, morbidity, or logistical demands of microsurgical reconstruction are limiting. However, the present study did not perform a cost-benefit analysis. Although a patient-specific plate may reduce intraoperative plate adaptation, it also introduces planning and manufacturing costs. Therefore, the present data cannot determine whether this workflow is economically advantageous compared with standard plates, conventional rib grafting, or vascularized flap reconstruction [22, 23].

### Limitations and future directions

This study has several limitations. First, the single-arm design without a control group is the major limitation of this study. It limits causal inference and prevents direct comparison with conventional reconstruction, standard plates, non-VSP rib grafting, or vascularized free-flap reconstruction. Therefore, the findings should not be interpreted as evidence of superiority, equivalence, or non-inferiority. A matched historical cohort was not included because previous cases did not have the same standardized VSP workflow, predefined radiographic landmark measurements, or serial CT-based graft-volume assessment, and because defect site and extent varied substantially.

Second, the sample size was modest and pragmatic, and the study was conducted in a single specialized center. The results may therefore reflect institutional expertise, patient selection, and learning-curve effects, limiting generalizability.

Third, the follow-up period was limited to 12 months. Although this interval is relevant for assessing early graft integration, mandibular continuity, occlusal stability, and early complications, it is insufficient to evaluate long-term graft remodeling, progressive resorption, stress shielding, screw loosening, dehiscence, plate fatigue, or outcomes beyond the early reconstructive phase.

Fourth, the radiographic analysis was landmark-based and was performed by a single examiner who could not be fully blinded because the reconstruction plate and graft were visible on postoperative CT imaging. Although intra-examiner reliability was excellent, inter-observer reliability was not assessed. Surface-based three-dimensional registration, mean surface deviation, and root-mean-square error analysis were not performed. Consequently, the study cannot provide full surface-based three-dimensional accuracy data and should be compared cautiously with contemporary VSP studies that use surface-registration methods.

Fifth, defects were categorized anatomically as body, body plus angle, and body plus angle plus ramus defects. A formal mandibular defect classification system such as Boyd or Cordeiro was not applied, which may limit comparability with other mandibular reconstruction studies. Bruxism and parafunctional habits were also not systematically assessed, although these factors may influence fixation loading, screw stability, graft remodeling, and plate loosening.

Sixth, patient satisfaction was assessed using a simple Likert scale rather than a validated quality-of-life instrument. Finally, no formal cost-benefit analysis was performed, and the cohort was intentionally restricted to selected benign, nonirradiated defects with adequate soft-tissue coverage. Therefore, the findings should not be extrapolated to malignant, irradiated, infected, composite, or soft-tissue-deficient defects. In addition, although corrections were applied to selected repeated comparisons, the analysis included multiple outcome domains and should therefore be interpreted as exploratory.

Future studies should include comparative cohorts, standardized defect classification, longer follow-up beyond 12 months, surface-based three-dimensional accuracy analysis, inter-observer reliability assessment, and formal cost evaluation. These studies are needed to determine the incremental value of VSP-assisted patient-specific plate reconstruction over conventional non-VSP rib grafting, standard plates, and vascularized reconstructive options [20, 23].

## Conclusion

Within the limitations of this prospective single-arm feasibility study, VSP-assisted mandibular reconstruction using a patient-specific reconstruction plate and split rib bundle graft was feasible in selected benign, nonirradiated mandibular defects with adequate soft-tissue coverage. The study demonstrated lower postoperative asymmetry indices, low landmark-based discrepancy values, favorable short-term occlusal findings, and 12-month graft survival in most patients. These findings should be interpreted as descriptive short-term outcomes and should not be considered evidence of superiority, equivalence, or non-inferiority compared with conventional non-VSP reconstruction, standard plates, or vascularized free-flap reconstruction. Comparative studies with standardized defect classification, longer follow-up, validated patient-reported outcome measures, inter-observer reliability assessment, and surface-based three-dimensional accuracy analysis are required.

## Data Availability

No datasets were generated or analysed during the current study.

## Abbreviations

ANOVA: Analysis of variance
CAD/CAM: Computer-aided design/computer-aided manufacturing
CI: Confidence interval
Co: Condylion
CT: Computed tomography
Go: Gonion
La: Lateral point
Me: Menton
Mf: Mental foramen
PSI: Patient-specific implant
SD: Standard deviation
SRBG: Split rib bundle graft
VSP: Virtual surgical planning
